# Supplementary material for: Rare disruptive mutations and their contribution to the heritable risk of colorectal cancer
Source: Nat Commun. 2016 Jun 22;7:11883. doi: 10.1038/ncomms11883 (PMC4917884; doi:10.1038/ncomms11883)
Supplement: Supplementary Information — Supplementary Figures 1-3, Supplementary Tables 1-8 and Supplementary Note 1. [file ncomms11883-s1.pdf]

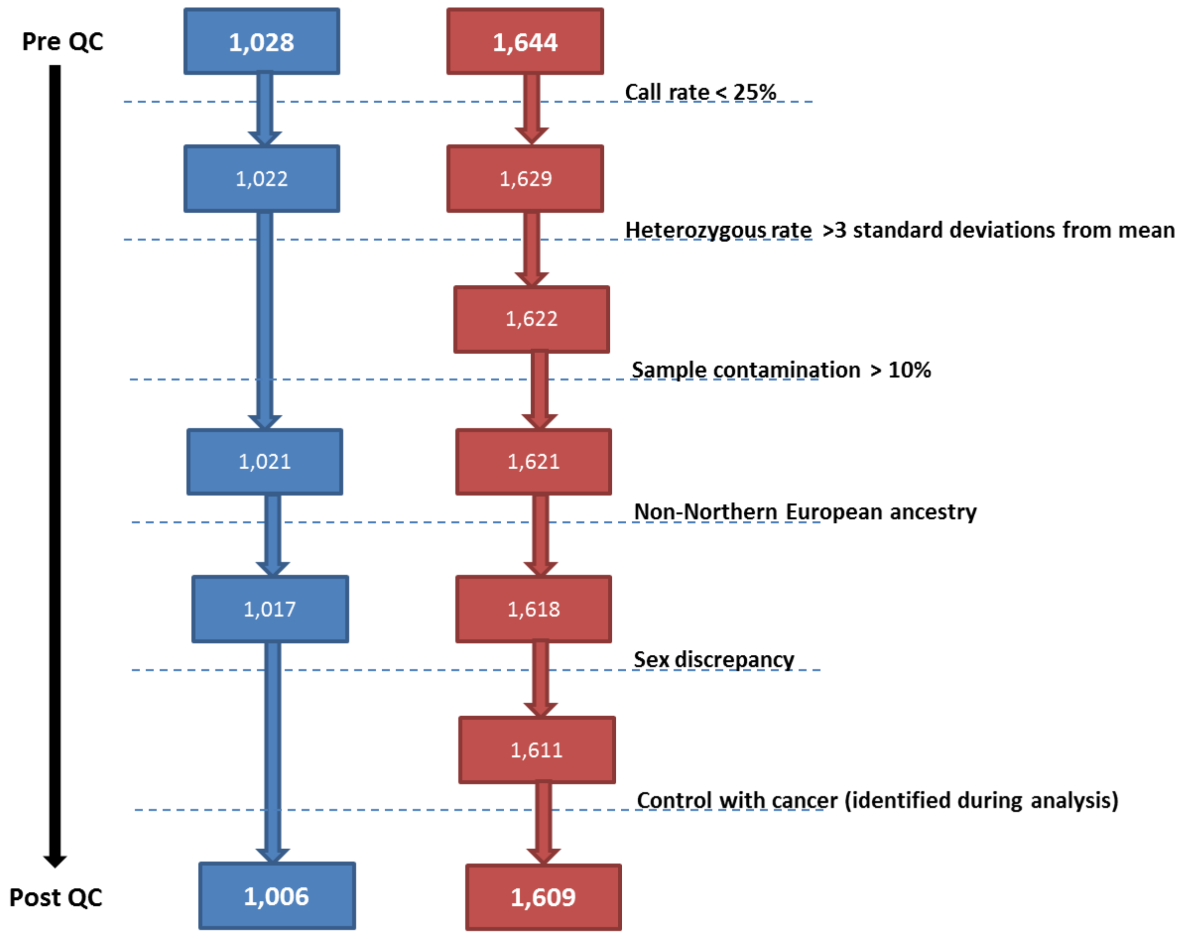

Supplementary Figure 1: Sample level quality control of whole exome sequencing in NSCCG cases and 1958BC controls.

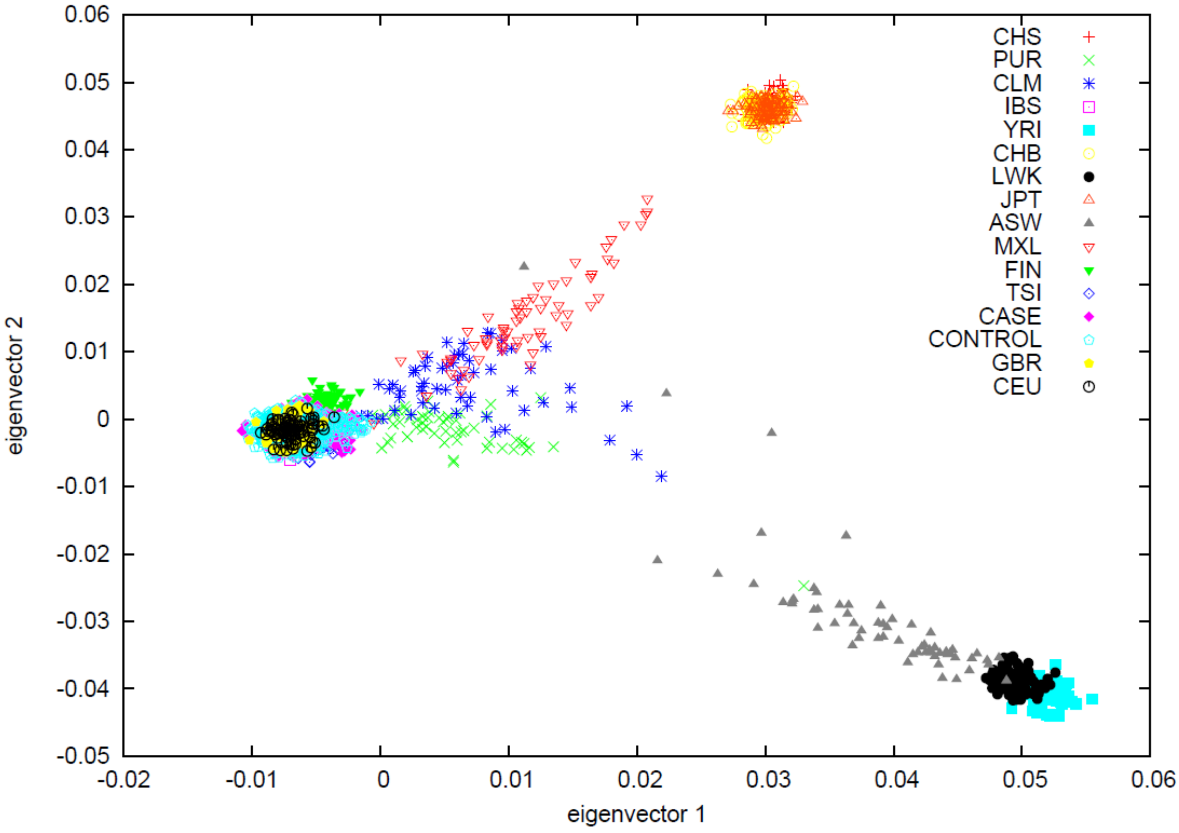

Supplementary Figure 2: Identification of individuals of non-European ancestry in NSCCG cases and 1958BC controls. The first two principal components of the analysis are plotted. NSCCG cases are plotted as pink diamonds, 1958BC controls plotted as light blue pentagons. Codes for HapMap populations are as per 1000Genomes**.**

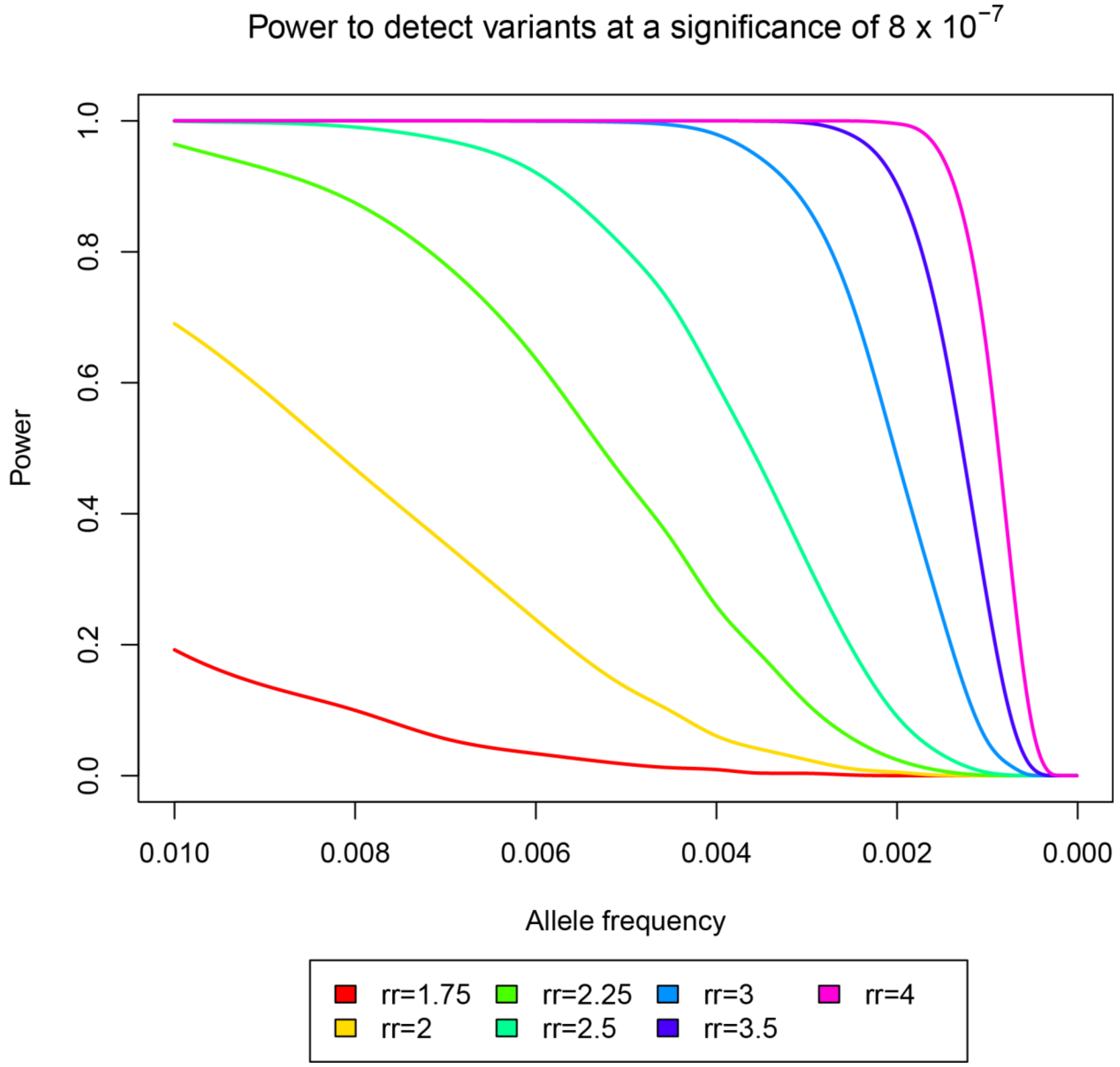

Supplementary Figure 3. The power to detect significant associations ($P \leq 8x10^{-7}$) that confer Relative risks of 1.75-4.

Category	Case (Average)	Control (Average)
Transition transversion ratio	3.003	2.957
Coverage ≥10x	85%	87%
Coverage ≥20x	73%	77%
Coverage ≥30x	59%	65%
Homozygous alternate	30,594	32,361
Heterozygous	53,319	55,069
SNP (in dbSNP)	75,494 (74,433)	78,717 (77,617)
Indel (in dbSNP)	8,419 (6,123)	8,713 (6,386)
Splice donor	27	27
Splice acceptor	24	24
Stop gained	39	40
Frameshift	96	98
Stop lost	11	11
Initiator codon	6	7
In-frame insertion	53	54
In-frame deletion	55	57
Missense	6,870	7,063
Splice region	1,701	1,756
Synonymous	8,015	8,269
Stop Retained	6	6
Coding sequence	8	9
Mature miRNA	3	3
5’ UTR	2,096	2,228
3’ UTR	16,593	16,934
Non-coding exon	2,645	2,728
Non-coding transcript	3,734	3,875
Intron	18,877	20,138
Upstream gene	10,036	10,539
Downstream gene	13,016	13,564

Supplementary Table 1: Whole exome sequencing and annotated variant statistics for NSCCG cases and 1958BC controls.

Supplementary Table 2: Characteristics of the NSCCG cases and 1958BC controls post quality control.

	Total	Male (%)	Type of colorectal cancer			Age (years)*		Number of relatives with CRC		
			Distal colon (%)	Proximal colon (%)	Rectal (%)	Average	Range	Average	Average first-degree	Amsterdam II (%)
**All Controls post-QC**	1,609	51.6				50	50			
**All Cases post-QC**	1,006	54.4	30.8	29.3	39.9	48.7	21-55	1.8	1.1	23.4
**Cases with pathogenic mutation in known gene**	143	58.7	27.3	57.3	15.4	43.7	21-55	2.8	1.3	53.8
All MMR	111	61.3	27.9	60.4	11.7	43.3	21-55	2.8	1.3	57.7
APC	19	47.4	15.8	47.4	36.8	43.7	32-55	3.0	1.3	47.4
MUTYH	9	44.4	33.4	44.4	22.2	49.9	39-55	1.4	1.0	0
POLE / POLD1	4	75	50	50	0	40.5	28-46	6.0	1.3	100
**Cases remaining**	863	53.7	31.5	24.6	43.9	49.5	23-55	1.6	1.1	18.4

* Age at diagnosis for cases; Age last censored for controls

Supplementary Table 3: Clinico-pathological characteristics of known CRC susceptibility gene mutation carriers.

Abbreviations:

1. M= male, F= female
2. FS= frameshift, ID= inframe deletion, MS= missence, SA= splice acceptor, SD= splice donor, SG= stop gain, SR= splice region
3. P= pathogenic, LP= likely-pathogenic, RF=risk factor
4. C= colon, P= proximal colon, R= rectal
5. TV= tubulovillous, TA= tubular adenoma, H= hyperplastic polyp
6. W/M= well moderate , P= poor , Mc= mucinous
7. MSI= microsatellite instability, MSS= microsatellite stable
8. F= father, M= mother, B= brother, S= sister, So= son, Da= daughter, U= uncle, A= aunt, GF= grandfather, GM= grandmother, Ne= nephew, Ni= Niece, GS= grandson, GD= granddaughter, C= cousin, GGF= great-grandfather, GGM= great-grandmother,H= half-relative, p= paternal, m= maternal, ICD=international classification of disease v9 (if not CRC)

Patient		Mutation						Diagnosis			Tumour			Family History	
Sex[1]	Age	Gene	Type[2]	c.DNA change	Protein change	InSight-ID	ClinVar[3]	Site	C(P)/R[4]	Polyps[5]	Grade[6]	Stage Dukes/TN	Other[7]	Relative/age	Amsterdam II
M	38	*APC*	FS	c.1612_1613insA	p.Asp539ArgfsTer21			1534	C(P)	Polyposis	W/M	B/T3-N0		F/52;	N
F	36	*APC*	FS	c.7452delA	p.Ser2485ValfsTer31			1531	C(P)	TA(7),TV(1)	W/M	A	Metachronous; MSS	B/40; F/49/ICD-150/162;	N
F	42	*APC*	FS	c.339_340insC	p.Met115TyrfsTer24			1539	C(NS)		NS	NS		M/70; F/50/ICD-172 mU/70; C/40; C/43	Y
F	43	*APC*	FS	c.2492_2493insA	p.Pro832ThrfsX12	APC_01122	P	1533/1541	C(D)	Polyposis	W/M	A;A;B	Synchronous	M/41; Multiple(>24)	Y
F	32	*APC*	FS	c.3921_3925delAAAAG	p.Glu1309AspfsTer4	APC_00006	P	1541	R	Polyposis	W/M	C/		M/40; B/33;	Y
M	48	*APC*	FS	c.3707_3708delCA	p.Gln1237GlufsTer2	APC_00383	P	1536	C(P)	Polyposis	W/M			F/75; p/GF/65	Y
M	37	*APC*	FS	c.3957delT	p.Val1320Ter			1541	R	Polyposis				M/33;	N
M	33	*APC*	FS	c.2182delA	p.Asn728IlefsTer33	APC_00675	P	1541	R	TA(1)	P			F/29; p/GF/37, p/GGM/48; p/GA; p/GA	Y
M	53	*APC*	FS	c.6011_6012insTT	p.Ser2005TyrfsTer40			1534	C(P)	TA(1),TV(1)	W/M			F/53;	N
M	45	*APC*	FS	c.2803_2804insA	p.Tyr935X	APC_00683	P	1534	C(P)	TA(Multiple)	P	B/T4-N0		M/36; S/47;	Y

M	33	*APC*	FS	c.3578_3579delAG	p.Gln1193LeufsTer14			1541	R	Polyposis	W/M	C/T1-N1		F/52; B/34;	Y
F	48	*APC*	SG	c.847C>T	p.Arg283X	APC_00001	P	1533	C(D)	Polyposis	P	C/T4-N2		B/40; M/65/ICD-180; m/GF; m/U	N
F	53	*APC*	SG	c.994C>T	p.Arg332Ter	APC_00082	P	1534	C(P)		W/M	C/T3-N2		B/45;	N
F	52	*APC*	SG	c.1213C>T	p.Arg405Ter	APC_00176	P	1534	C(P)	TA(Multiple)	W/M	A/T2-N0		M/74; m/GF/65; m/A/55; m/A/56	N
M	51	*APC*	SG	c.994C>T	p.Arg332X	APC_00082	P	1541	R	Polyposis	W/M	C/T3-N1		F/58;	N
M	48	*APC*	SG	c.3593C>G	p.Ser1198Ter	APC_00062	P	1534	C(P)	Polyposis	W/M	C/T4-N1		M/45;	N
F	51	*APC*	SG	c.6620C>G	p.Ser2207Ter			1531	C(P)	TA(1),TV(1)		C/		F/70; B/50; m/A	N
F	40	*APC*	SG	c.637C>T	p.Arg213X	APC_00034	P	1541	R	TV(multiple)	W/M			M/31; S/33; p/GM/40; p/U/25; p/A/60; p/C/14; p/C/15; p/GU; p/GU	Y
F	48	*APC*	SG	c.994C>T	p.Arg332Ter	APC_00082	P	1541	R	TV(multiple)	P	C/T4-N2		S/43; m/GF/70; Ne/23; Ne/29; Ni/24; Ni/25	Y
M	40	*MLH1*	FS	c.405_406insA	p.Ala137SerfsTer4			1531	C(P)	TV(2)	NS	NS		B/42; F/51/ICD-188;	N
M	29	*MLH1*	FS	c.1344_1345delGG	p.Asp450TyrfsTer28			1536	C(P)		W/M	C/T3-N1		M/34; mGM/34	Y
M	44	*MLH1*	FS	c.1190delT	p.Leu397ArgfsTer4	MLH1_01088	P	1532	C(D)		P	C/T3-N1		M/46; m/U/44; m/C/25	Y
M	42	*MLH1*	FS	c.345_346insA	p.Thr116AsnfsTer6	MLH1_00837	P	1536	C(P)		P	B/T4-N0	Loss of MLH1/PMS2	F/53; p/A; p/A; m/GM	Y
M	37	*MLH1*	FS	c.1451delA	p.Asp484ValfsTer7			1530	C(P)		P	B/T3-N0		F/43; p/GF/40	Y
F	50	*MLH1*	FS	c.1451delA	p.Asp484ValfsTer7			1536	C(P)			NS		F/48;	N
M	50	*MLH1*	FS	c.1757delC	p.Met587CysfsTer4	MLH1_01191	P	1532	C(D)		W/M	B/T3-N0		F/40; S/39 p/U/45; p/GF/45	Y
M	48	*MLH1*	FS	c.1484delC	p.Arg497GlyfsTer11	MLH1_01121	P	1533	C(D)		W/M	C/T3-N2		M/59; m/HS/36	Y
M	35	*MLH1*	FS	c.206delG	p.Glu71LysfsTer21			1534	C(P)		P	C/T3-N2		F/60; M/58; B/29;	Y
M	52	*MLH1*	FS	c.382delG	p.Ala128GlnfsTer8	MLH1_01027	P	1533	C(D)			NS		M/40;	N
F	47	*MLH1*	FS	c.1132_1133insT	p.Tyr379LeufsTer16			1536	C(P)		W/M	B/T3-N0		S/39; S/45; M/45/ICD-183; m/A/32; m/A/72; C/31	Y
F	37	*MLH1*	FS	c.345_346insA	p.Thr116AsnfsTer6	MLH1_00837	P	1537	C(D)		W/M	C/T3-N1	MSI	F/57; mGM	Y
M	48	*MLH1*	FS	c.206delG	p.Glu71LysfsTer21			1540	R		W/M	B/T3-N0		S/38; M/73/ICD-188; A/59	N

F	52	*MLH1*	FS	c.1451delA	p.Asp484ValfsTer7			1531	C(P)					M/39; m/GM/39; A/70	Y
F	48	*MLH1*	FS	c.1377_1378delAG	p.Lys461GlufsTer17	MLH1_00844	P	1531	C(P)	TA(1),TV(1)				M/40; B/28; m/GM; m/U/34; m/U/68; m/A, m/GGM; m/GA	Y
M	21	*MLH1*	FS	c.409delG	p.Ala137ProfsTer23			1534	C(P)	TV(1)	W/M	B/T3-N0	MSI	M/31; S/15/ICD-155/157 mGF; mGGF	Y
M	52	*MLH1*	MS	c.350C>T	p.Thr117Met	MLH1_01492	P	1533	C(D)		P	C/T4-N2		M/50; S/50; U; U	N
M	43	*MLH1*	MS	c.350C>T	p.Thr117Met	MLH1_01492	P	1531	C(P)		W/M	B/T3-N0		M/82; m/GM/70; GGF/50	Y
F	41	*MLH1*	MS	c.199G>A	p.Gly67Arg	MLH1_00966	P	1534	C(P)		W/M	C/T4-N1	Loss of MLH1/PMS2	F/30;	N
F	55	*MLH1*	MS	c.350C>T	p.Thr117Met	MLH1_01492	P	1536	C(P)		W/M	B/T3-N0		F/84;	N
F	55	*MLH1*	MS	c.554T>G	p.Val185Gly	MLH1_00268	P	1533	C(D)		P	C/T4-N1		F/41; p/GM; p/U; p/A; p/A	Y
F	38	*MLH1*	MS	c.380G>A	p.Arg127Lys	MLH1_01023	LP	1534	C(P)		P	C/T3-N1	MSI	M/53; m/U/49; m/GF/66; m/GM/62	Y
M	39	*MLH1*	MS	c.677G>A	p.Arg226Gln	MLH1_01558	P	1531	C(P)		P	B/T4-N0		M/58; S/40/ICD-183; A; U; GM	Y
M	34	*MLH1*	MS	c.350C>T	p.Thr117Met	MLH1_01492	P	1531	C(P)	TA(1)	P	C/T4-N2		M/33; mGM/40; C/35	Y
F	39	*MLH1*	MS	c.199G>A	p.Gly67Arg	MLH1_00966	P	1530	C(P)	TA(1)	P			F/40; B/34; S/30/ICD-182;	Y
M	44	*MLH1*	MS	c.200G>A	p.Gly67Glu	MLH1_00110	P	1537	C(D)	TA(1),TV(1)	W/M			F/43; p/GF/62; p/A/40; p/A/59; p/C/24; p/C/37; p/C/46; p/C/50	Y
F	46	*MLH1*	MS	c.350C>T	p.Thr117Met	MLH1_01492	P	1536	C(P)		P			F/47; S/33;	Y
F	47	*MLH1*	SA	c.546-2A>G	p.Arg182SerfsTer6	MLH1_00256	P	1536	C(P)		P	B/T4-N0		F/73;	N
M	41	*MLH1*	SA	c.208-2A>G		MLH1_00122	P	1533	C(D)		W/M	C/T4-N1		F/51; m/GF/70	N
M	52	*MLH1*	SA	c.1668-1G>A	p.Ser556ArgfsTer14	MLH1_01166	LP	1541	R		W/M	B/T3-N0		M/68;	N
M	46	*MLH1*	SA	c.1668-1G>A	p.Ser556ArgfsTer14	MLH1_01166	LP	1536	C(P)		W/M	B/T3-N0		M/47; S/39; GM	Y
F	42	*MLH1*	SA	c.381-2A>G	p.Arg127_Ala128del	MLH1_00217	LP	1534	C(P)	H(1)	P	C/T3-N1		F/27;	N
F	32	*MLH1*	SA	c.1668-1G>A	p.Ser556ArgfsTer14	MLH1_01166	LP	1531	C(P)	TV(1)	W/M	B/T3-N0	MSI	F/34;	N
M	54	*MLH1*	SA	c.1668-1G>A	p.Ser556ArgfsTer14	MLH1_01166	LP	1540	R	TV(2)	W/M	B/T4-N0		F/54; S/47; p/U; p/U	Y
M	47	*MLH1*	SD	c.588+1G>T	p.Arg182SerfsTer6	MLH1_01321	P	1530	C(P)		W/M	B/T3-N0		M/55; S/55; m/U/55; m/C/40	Y

M	51	*MLH1*	SD	c.2103+1G>A		MLH1_00765	P	1536	C(P)	TA(2)	P	B/T3-N0		B/38; M/35/ICD180/151	N
F	43	*MLH1*	SG	c.2135G>A	p.Trp712Ter	MLH1_00799	P	1530	C(P)		W/M	B/T3-N0		F/37; D/11/ICD-191; p/A/41; p/U/30; p/GF/55; C/43, p/GGM, GA; GU	Y
M	36	*MLH1*	SG	c.1636A>T	p.Lys546Ter			1534	C(P)		W/M	B/T3-N0		F/44; p/GM	Y
F	43	*MLH1*	SG	c.1849A>T	p.Lys617Ter			1531/1541	C(NS)		W/M	B;A/T3-N0;T2-N0	Synchronous	F/39; S/38; p/U	Y
F	34	*MLH1*	SG	c.979C>T	p.Gln327Ter			1537	C(D)		P	C/T4-N2		F/34; S/36; A/27; GM	Y
F	51	*MLH1*	SG	c.901C>T	p.Gln301Ter	MLH1_00407	P	1534/1536	C(P)	TV(1)	W/M	A;A/T1-N0;T2-N0	Synchronous	F/59; p/A/43; p/GF/48; p/C/35	Y
M	39	*MLH1*	SG	c.378C>G	p.Tyr126Ter	MLH1_00209	P	1534	C(P)		P	C/	MSI	M/47; m/U/48; m/U/39, m/GF/53; m/GA/53; m/GA/53; m/GU/40	Y
F	43	*MLH1*	SG	c.676C>T	p.Arg226Ter	MLH1_00285	P	1534	C(P)	TA(2)	W/M	C/T4-N1		M/52; S/40; m/GM	Y
F	35	*MLH1*	SR	c.116+5G>C	p.Cys39Trpfs*11	MLH1_01083	P	1533	C(D)		W/M	C/T3-N1;T2-N1		F/39;	N
M	53	*MLH1*	SR	c.882C>T	p.His264LeufsTer2	MLH1_00382	P	1534	C(P)		W/M	B/T3-NO		F/70;	N
M	51	*MLH1*	SR	c.116+5G>C	p.Cys39Trpfs*11	MLH1_01083	P	1531	C(P)		W/M	C/T3-N1		F/57;	N
M	39	*MSH2*	FS	c.967_968insCTCA	p.Gln324HisfsTer10			1534	C(P)	TA(1)	P	B/T3-N0		F/40; p/GM/77	Y
M	43	*MSH2*	FS	c.1699_1703delAAAAC	p.Lys567ArgfsTer3	MSH2_00487	P	1531	C(P)	TA(1)	NS	B/T3-N0	MSS	M/32; F/73; m/FAMILY	Y
F	39	*MSH2*	FS	c.628_629delAT	p.Met210GlyfsTer21			1534	C(P)		P	C/T4-N2		F/59;	N
M	47	*MSH2*	FS	c.2501_2507delCTAATTT	p.Asn835LeufsTer4	MSH2_01168	P	1534	C(P)		NS	C		F/65;	N
F	45	*MSH2*	FS	c.2100delA	p.Glu701LysfsTer9			1534	C(P)		W/M	C/T3-N2		F/47;	N
M	49	*MSH2*	FS	c.2501_2507delCTAATTT	p.Asn835LeufsTer4	MSH2_01168	P	1533	C(D)		P	B/T4-N0		M/45; m/U/34; m/U/50; m/C/54	Y
F	47	*MSH2*	FS	c.1577delC	p.Cys527ValfsTer16	MSH2_00432	P	1541	R		W/M	A/T1-N0		F/85; B/40; B/53; S/45/ICD-182; p/A/66; p/GM/60	Y
M	42	*MSH2*	FS	c.1699_1703delAAAAC	p.Lys567ArgfsTer3	MSH2_00487	P	1533	C(D)		W/M	A/T2-N0		B/37; F/56/ICD-188/189	Y
M	40	*MSH2*	FS	c.1577delC	p.Cys527ValfsTer16	MSH2_00432	P	1530	C(P)		W/M	C/T3-N1		M/59; m/GF	Y
F	46	*MSH2*	FS	c.1249_1252delGTTA	p.Val417TyrfsTer20			1537	C(D)		NS	NS		F/45; B/43; S/41; p/U/55; p/U/55	Y

F	44	*MSH2*	FS	c.838delT	p.Leu280TyrfsTer12			1541	R		W/M	NS		B/27; M/54/ICD-182 m/U/47	N
M	37	*MSH2*	FS	c.2501_2507del CTAATTT	p.Asn835LeufsTer4	MSH2_01168	P	1536	C(P)		W/M	A/T4-N0		F/50; B/32; S/29; GF/40	Y
M	45	*MSH2*	FS	c.161delC	p.Arg55GlyfsTer9	MSH2_00009	P	1534	C(P)				MSI	F/77; B/27; B/48/ICD-182 m/GF/45	Y
M	38	*MSH2*	FS	c.1985_1986del AG	p.Gln662HisfsTer13	MSH2_00528	P	1534	C(P)		P	C/T4-N1		M/59; m/U/35; m/GF/60: m/GU/38	Y
M	45	*MSH2*	FS	c.1226_1227del AG	p.Gln409ArgfsTer7	MSH2_01311	P	1531	C(P)	TA(2)	P	B/T3-N0	MSI	F/51; p/A; p/A; p/C	Y
M	54	*MSH2*	FS	c.1699_1703del AAAAC	p.Lys567ArgfsTer3	MSH2_00487	P	1531	C(P)	TA(3)				F/55; S/44; S/51/ICD-180;	Y
F	27	*MSH2*	FS	c.2502_2508del TAATTTC	p.Asn835LeufsTer4	MSH2_01168	P	1537	C(D)	TV(5)	W/M	A/T2-N0		M/48; m/A/20; m/A/49; m/GM/75; m/C/24	Y
F	31	*MSH2*	ID	c.1786_1788del AAT	p.Asn596del	MSH2_01381	P	1536	C(P)		P	C	MSI	F/31; m/U/31; m/U/50; m/A/60; m/C/30; m/C/47; m/C/50	Y
M	50	*MSH2*	ID	c.1786_1788del AAT	p.Asn596del	MSH2_01381	P	1536	C(P)		P	C/T3-N1		S/30; B/47; F/74/ICD-172; M/54/ICD-157; m/U/31; m/U/50; m/A/54; m/A/60; m/C/31; p/U/73	Y
F	39	*MSH2*	MS	c.560T>C	p.Leu187Pro	MSH2_00169	P	1531	C(P)		P	C/T3-N2	Loss of MSH2/6	M/60; m/A/51; m/U/62	Y
F	54	*MSH2*	SA	c.1915C>T + c.2211-1G>T	p.His639Profs*6	MSH2_00537	LP	1532	C(D)		W/M	B/T3-N0		M/52; m/A	N
M	30	*MSH2*	SG	c.1009C>T	p.Gln337Ter	MSH2_00271	P	1532	C(D)		W/M	B/T3-N0	MSI	F/44; p/GF/50	Y
F	55	*MSH2*	SG	c.1801C>T	p.Gln601Ter	MSH2_00524	P	1530/1537	C(P)	TA(1),TV(1)	W/M	C;B/T3-N2;T3-N0	Synchronous	F/61, S/41, So/30; GF; U; A; C/30, Ni/19	Y
M	27	*MSH2*	SG	c.1165C>T	p.Arg389Ter	MSH2_00311	P	1541	R		W/M	B/T4-N0		M/41;	N
M	52	*MSH2*	SG	c.2285T>A	p.Leu762Ter			1536	C(P)		W/M	C/T3-N1		F/55; B/40; C; C	Y
M	46	*MSH2*	SG	c.1216C>T	p.Arg406Ter	MSH2_00312	P	1533	C(D)		P	C/T3-N1		M/65; m/U; m/GF; m/A; m/A	Y
M	55	*MSH2*	SG	c.2563C>T	p.Gln855Ter			1541	R		W/M	NS		F/52;	N
M	38	*MSH2*	SG	c.1861C>T	p.Arg621Ter	MSH2_01323	P	1539	C(NS)		W/M	NS	Metachronous	F/39; M/78; m/HB/56; m/A; m/U	Y

M	48	*MSH2*	SG	c.1861C>T	p.Arg621Ter	MSH2_01323	P	1533	C(D)		W/M	C/T4-N2		B/31; B/50; M/68/ICD-162;	N
M	53	*MSH2*	SG	c.1861C>T	p.Arg621Ter	MSH2_01323	P	1533/1534	C(NS)		W/M	B/T4-N0	Synchronous	F/62;	N
M	45	*MSH2*	SG	c.970C>T	p.Gln324Ter	MSH2_01304	P	1534	C(P)		P	C/T4-N2		M/28; mGM/45	Y
M	49	*MSH2*	SG	c.754C>T	p.Gln252Ter	MSH2_00197	P	1531	C(P)					F/40;	N
F	37	*MSH2*	SG	c.1566C>G	p.Tyr522Ter	MSH2_00981	P	1536	C(P)		W/M	C/T4-N1	MSI	F/57;	N
M	26	*MSH2*	SG	c.1351C>T	p.Gln451Ter			1532	C(D)		W/M	B/T3-N0		M/39;	N
F	47	*MSH2*	SG	c.2563C>T	p.Gln855Ter			1531	C(P)		W/M	C/T3-N2		M/35; mA/50; C/45; C/53	Y
M	47	*MSH2*	SG	c.2563C>T	p.Gln855Ter			1532	C(D)		P	C/T3-N1		M/48; B/48; B/54; A/47; A/49; C/47; C/50; C/54	Y
F	47	*MSH2*	SG	c.1165C>T	p.Arg389Ter	MSH2_00311	P	1534	C(P)	TA(1)				B/43; M/44/ICD-157; m/A/52; m/GF/53; m/C/47; m/C/63	Y
F	53	*MSH2*	SG	c.1165C>T	p.Arg389Ter	MSH2_00311	P	1541	R	TA(1),TV(1)	W/M	A/T1-N0		F/38; S28; S39; p/GF; p/C	Y
F	55	*MSH2*	SG	c.1216C>T	p.Arg406Ter	MSH2_00312	P	1531	C(P)	TV(1)	W/M	A/T2-N0	MSI	B/49; B/32; M/31/ICD-174;	N
M	34	*MSH2*	SG	c.1738G>T	p.Glu580Ter	MSH2_00478	P	1531	C(P)	TV(1)	W/M			M/43; mGM/54	Y
M	47	*MSH2*	SG	c.1165C>T	p.Arg389Ter	MSH2_00311	P	1541/1536	R	TV(1)	W/M	A/T2-N0	Synchronous	F/70;	N
F	31	*MSH2*	SR	c.942+3A>T	p.Val265_Gln314del	MSH2_00260	P	1533	C(D)		P	B/T4-N0		F/47; p/A/65	Y
M	45	*MSH2*	SR	c.942+3A>T	p.Val265_Gln314del	MSH2_00260	P	1534	C(P)		W/M	B/T4-N2		B/38; M/50/ICD-193; U/42; U/48; A; C/54	Y
M	50	*MSH2*	SR	c.792+1G>A	p.Ile216_Gln264del	MSH2_00224	P	1534	C(P)		W/M	C/T3-N1		B/33; F/72/ICD-151	N
M	29	*MSH2*	SR	c.942+3A>T	p.Val265_Gln314del	MSH2_00260	P	1534	C(P)		W/M	B/T4-N0		M/45; B/32;	Y
M	43	*MSH2*	SR	c.942+3A>T	p.Val265_Gln314del	MSH2_00260	P	1534	C(P)	TV(1)	W/M	C/T3-N2		M/47;	N
M	54	*MSH6*	FS	c.3253_3254insC	p.Phe1088LeufsTer5	MSH6_00201	P	1541	R		W/M	C/T3-N1		M/65; S/20/ICD-180;	N
M	49	*MSH6*	FS	c.674_675insTG	p.Glu226ValfsTer2			1541	R		NS	C/T3-N2		F/67;	N
F	38	*MSH6*	FS	c.3475_3476insA	p.Tyr1159Ter	MSH6_00612	P	1539	C(NS)		W/M	C/T3-N1	Loss of MSH2/6	F/57; M/68/ICD-174; m/C/36	N

M	34	*MSH6*	FS	c.1503_1504insATATCCAAGTATG	p.Arg507IlefsTer4			1533	C(D)					F/49;	N
F	54	*MSH6*	FS	c.3253_3254insC	p.Phe1088LeufsTer5	MSH6_00201	P	1539	C(NS)		W/M	B/T4-N0		F/65; B/59/ICD-172; M/46/180; A	N
F	48	*MSH6*	FS	c.1635_1636delAG	p.Glu546GlyfsTer16	MSH6_00407	P	1536; 1531/1533	C(P)	H(Multiple), TV(1)	W/M	B/T3-N0	Metachronous	F/54; C	N
F	53	*MSH6*	MS	c.2057G>A	p.Gly686Asp	MSH6_00785	LP	1534/1537/1541	C(NS)		W/M	C;C;B/1534:T4-N1;1537:T3-N1;1541:T3-N0	Synchronous; MSI	F/59; m/GM/52; m/GF/85; A	N
F	32	*MSH6*	SA	c.3439-1G>T		MSH6_00713	LP	1541	R		W/M	A/T2-N0		M/50;	N
M	52	*MSH6*	SA	c.3439-1G>T		MSH6_00713	LP	1534	C(P)		W/M	B/T3-N0		F/71; M/68/ICD-183; m/A/84	N
M	46	*MSH6*	SG	c.718C>T	p.Arg240Ter	MSH6_00612	P	1534/1541	C(P)	TV(1)	W/M	B;C/T3-N1/T3-N0	Synchronous	F/76; S/48/ICD-182;	Y
M	49	*MSH6*	SG	c.2731C>T	p.Arg911Ter	MSH6_00071	P	1531	C(P)		P	B/T3-N0		F/72; pU/70	Y
M	28	*MSH6*	SG	c.694C>T	p.Gln232Ter	MSH6_00366	P	1541	R		W/M	B/T3-N0		M/54;	N
M	38	*MSH6*	SG	c.3140G>A	p.Trp1047Ter			1530	C(P)	TA(1)	W/M	B/T4-N0	MSI	M/48;	N
F	55	*MUTYH*	MS	c.536A>G, c.1187G>A	p.Tyr179Cys + p.Gly396Aspl		P	1533	C(D)	H(Multiple)	W/M	C/T4-N2		M/52; GA	N
F	39	*MUTYH*	MS	c.1187G>A + c.1187G>A	p.Gly396Asp	MUTYH_00075	P	1534	C(P)		W/M	B/T4-N0		S/33; F/55/ICD-188; M/42/ICD-183;	N
F	48	*MUTYH*	MS	c.536A>G + c.690G>A	p.Tyr179Cys + p.Val179_Gln230del		P	1532/1534	C(D)	Polyposis	W/M	C/T4-N1;T1-N0	Synchronous	M/76;	N
F	53	*MUTYH*	MS	c.1214C>T + c.1214C>T	p.Pro405Leu	MUTYH_00012	P	1541	R					S/38;	N
M	51	*MUTYH*	MS	c.536A>G + c.536A>G	p.Tyr179Cys	MUTYH_00012	P	1531	C(P)		W/M	B/T3-N0		F/83; m/A/85	N
M	48	*MUTYH*	MS	c.1187G>A + c.1187G>A	p.Gly396Asp	MUTYH_00075	P	1541	R		W/M	C/T3-N2		F/72;	N
M	54	*MUTYH*	MS	c.536A>G, c.1187G>A	p.Tyr179Cys + p.Gly396Aspl		P	1534	C(P)	TA(Multiple)	P	C/T3-N1	MSS	M/73; S/65/ICD-174;	N

M	54	*MUTYH*	MS	c.1187G>A + c.1187G>A	p.Gly396Asp	MUTYH_00075	P	1534	C(P)	H(Multiple), TV(1)	W/M	B/T3-N0		B/49;	N
F	47	*MUTYH*	MS	c.536A>G, c.1187G>A	p.Tyr179Cys + p.Gly396Aspl		P	1533	C(D)	TV(1)	W/M	C/T4-N1		S/45; m/GM/65; m/GA/53	N
F	21	*PMS2*	FS	c.17_18delGC	p.Ser6IlefsTer7			1539	C(NS)		NS	NS		M/37; m/GF/80	Y
M	35	*PMS2*	FS	c.736_740delCCCCCinsGTGTGTGAAG	p.Pro246Cysfs*3	PMS2_00187	P	1534	C(P)		W/M	B/T3-N0		M/60;	N
M	48	*PMS2*	FS	c.63_75del, c.78_107del	p.Val23LeufsTer2			1534	C(P)		W/M	C/T3-N2		F/56;	N
F	49	*PMS2*	MS	c.137G>T	p.Ser46Ile		LP	1537	C(D)		W/M	B/T4-N0		F/56 m/A/62; m/GF	N
M	45	*PMS2*	MS	c.137G>T	p.Ser46Ile		LP	1536	C(P)		W/M	B/T3-N0	Loss of PMS2	F/44; M/65/ICD-182 m/U/60	Y
M	33	*PMS2*	MS	c.137G>T	p.Ser46Ile		LP	1536	C(P)	Polyposis	W/M	B/T3-N0		B/34;	N
M	28	*POLD1*	MS	c.1433G>A	p.Ser478Asn	POLD1_000001	RF	1537	C(D)	TA(1),TV(1)	W/M			F/44; p/GM/36, GA/63	Y
M	46	*POLE*	MS	c.1270C>G	p.Leu424Val	POLE_000001	RF	1534	C(P)	TA(Multiple)	W/M	B/T3-N0		F/65; p/U/28; p/U/45; p/A/40; p/A/58; p/gf/47; p/C/25; p/C/38; p/C/46; p/C/54	Y
M	43	*POLE*	MS	c.1270C>G	p.Leu424Val	POLE_000001	RF	1530	C(P)	H(2),TA(1),TV(2)	W/M	C/T4-N2		M/60; S/54;	Y
F	45	*POLE*	MS	c.1270C>G	p.Leu424Val	POLE_000001	RF	1538	C(NS)	TA(1),TV(1)	W/M	C/T3-N1		F/28; p/FAMILY	Y

Supplementary Table 4: Clinico-pathological characteristics of candidate mutation carriers.

Abbreviations:

1. M= male, F= female
2. FS= frameshift, SG= stop gain
3. P= pathogenic, LP= likely-pathogenic, RF=risk factor
4. C= colon, P= proximal colon, R= rectal
5. TV= tubulovillous, TA= tubular adenoma, H= hyperplastic polyp
6. W/M= well moderate , P= poor , Mc= mucinous
7. MSS= microsatellite stable
8. F= father, M= mother, B= brother, S= sister, So= son, Da= daughter, U= uncle, A= aunt, GF= grandfather, GM= grandmother , p= paternal, m= maternal, ICD=international classification of disease v9 (if not CRC)

	Patient		Mutation					Diagnosis			Tumour			Family History	
**Series**	**Sex[1]**	**Age**	**Gene**	**Type[2]**	**c.DNA change**	**Protein change**	**ClinVar[3]**	**Site**	**C(P)/R[5]**	**Polyps[7]**	**Grade[8]**	**Stage Dukes/TN[9]**	**Other[10]**	**Relative(age)**	**Amsterdam II**
Exomes	M	48	*IL12RB1*	SG	c.94C>T	p.Gln32Ter	P	1536	C(P)			B/T3-N0		M/30	N
Exomes	F	44	*IL12RB1*	SG	c.1624C>T	p.Gln542Ter		1531	C(P)					F/38; p/GF	Y
Exomes	M	53	*IL12RB1*	SG	c.1624C>T	p.Gln542Ter		1540	R	H(1),TA(1)	W/M	C/T2-N1		M/65; mA/35	Y
Exomes	F	49	*IL12RB1*	SG	c.1624C>T	p.Gln542Ter		1541	R					M/63; D/9/ICD-2025	N
WGSET	F	61	*IL12RB1*	SG	c.1624C>T	p.Gln542Ter						B		M, mA	N
WGSET	F	66	*IL12RB1*	SG	c.1624C>T	p.Gln542Ter		1534	C(P)		P	B/T3-N0	MSS	M/67	N
Exomes	M	55	*LIMK2*	FS	c.1711-1712insC	p.Gly574ArgfsTer12		1531	C(P)	TA(1)	W/M	A/T2-N0		F/66; pGM; S/48/ICD-193; M/76/ICD-162	N
Exomes	M	51	*LIMK2*	FS	c.1711-1712insC	p.Gly574ArgfsTer12		1541	R	H(1)	W/M	C/T3-N1		F/70	N
Exomes	F	54	*LIMK2*	FS	c.1711-1712insC	p.Gly574ArgfsTer12		1536/1530	C(P)		P	C/T4-N2	MSS	F/75; pA/75	N
Exomes	M	55	*LIMK2*	FS	c.1711-1712insC	p.Gly574ArgfsTer12		1533	C(D)		W/M		MSS	M/82; mC; A/ICD-174/179	N
Exomes	F	55	*LIMK2*	FS	c.1711-1712insC	p.Gly574ArgfsTer12		1540	R		W/M	C/T4-N1	MSS	S/56/ICD-153/183; F/70; S/64/ICD-183	N
WGSET	F	46	*LIMK2*	FS	c.2049_2050insA	p.Cys582LeufsTer4		1541	R			C/T3-N1	MSS	pGM/61; M/69/ICD-174;	N

WGSET	M	58	*LIMK2*	FS	c.2049_2050insA	p.Gln684ThrfsTer16				TA(2)		A		B/49; mU/68; mA/ICD-188	N
Exomes	M	43	*MRE11A*	FS	c.1066delC	p.His356ThrfsTer34		1541	R	TA(2)	P	C/T4-N2		M/61	N
Exomes	M	55	*MRE11A*	SA	c.21-6_26delATATAGTGATGA	p.Leu7fsTer18	LP	1540	R		W/M	C/T3-N2	MSS	F/62	N
Exomes	F	51	*MRE11A*	SG	c.1726C>T	p.Arg576Ter	P	1541	R	H(1)	W/M	B/T3-N0		F/78; U/75	N
Exomes	M	44	*NTHL1*	SG/SG	c.268C>T/c.859C>T	p.Gln90Ter/p.Gln287Ter	P	1541	R	TV(1)	W/M(Mc)	C/T3-N1		F/72; pGM/70	Y
Exomes	F	48	*POLE2*	FS	c.1406dupT	p.Leu469PhefsTer17		1541	R					S/35; F/75/ICD-191	N
Exomes	M	52	*POLE2*	FS	c.1406dupT	p.Leu469PhefsTer17		1533	C(D)		P	C/T3-N1		F/67; S/64; GF	N
Exomes	M	52	*POLE2*	FS	c.1406dupT	p.Leu469PhefsTer17		1537/1533	C(D)					F/67	N
WGSET	M	47	*POLE2*	FS	c.1406dupT	p.Leu469PhefsTer17						C		M/38; mU; mU/ICD-151	Y
WGSET	F	61	*POLE2*	FS	c.1406dupT	p.Leu469PhefsTer17		153/183				C		S/61	N
Exomes	M	54	*POT1*	FS	c.1851_1852delTA	p.Asp617GlufsTer9	RF	1541	R		W/M			M/47;ICD-202/76	N
Exomes	F	54	*POT1*	SG	c.1087C>T	p.Arg363Ter		1536	C(P)					B/40; M/36; mA/60; mU/58; GM/64	Y
WGSET	M	54	*POT1*	SG	c.219_220insA	p.Asn75LysfsTer16		1541	R		W/M	C/T4-N1	MSS	M/50	N

Pathway	Q	Genes																				
		SCAMP2	GOLGA5	STEAP2	MRE11A	POLE2	POT1	MSH6	MSH2	MLH1	PMS2	ZBTB38	ERCC3	MYO6	NRIP1	ERCC2	EIF2B4	EIF2B3	EIF2AK3	TP53	ERCC6	POLG
**DNA_REPLICATION**	**0.08**	**-**	**-**	**-**	**1.1**	**1.5**	**1.1**	**2.8**	**6.2**	**6.2**	**1.1**	**-**	**-**	**-**	**-**	**-**	**-**	**-**	**-**	**-**	**-**	**-**
GOLGI_VESICLE_TRANSPORT	0.13	1.1	1.1	1.0	-	-	-	-	-	-	-	-	-	-	-	-	-	-	-	-	-	-
POSITIVE_REGULATION_OF_TRANSCRIPTION_FROM_RNA_POLYMERASE_II_PROMOTER	0.12	-	-	-	-	-	-	-	-	-	-	0.7	1.0	1.1	1.1	0.7	-	-	-	1	-	-
DNA_DEPENDENT_DNA_REPLICATION	0.11	-	-	-	-	-	-	2.8	6.2	6.2	1.1	-	-	-	-	-	-	-	-	-	-	-
BASE_EXCISION_REPAIR	0.12	-	-	-	-	-	-	2.8	6.2	-	-	-	-	-	-	-	-	-	-	0.7	1.7	0.7
CELLULAR_RESPONSE_TO_STIMULUS	0.15	-	-	-	-	-	-	-	-	-	-	-	-	-	-	-	0.7	1.1	0.7	0.7	-	-

Supplementary Table 5: Gene Set Enrichment Analysis (GSEA) of GO Biological Process ontologies shows a significant association for colorectal cancer with DNA replication. Shown are the genes contributing to the leading edge of all pathways with a GSEA Q value < 0.25. Only for the DNA_REPLICATION gene set was a significant GSEA shown (i.e. Q value < 0.1). The genes displayed are those that contribute to the leading edge of the gene set, value in each cell is the –log10(P_{T1}) used in the ranking for GSEA.

Supplementary Table 6: Occurrence of Class 2 co-mutations in cases for known CRC predisposition genes

Gene	*BMPR1A*	*POLE*	*MSH6*	*MSH2*	*MLH1*	*APC*	*POLD1*	*PMS2*	*PTEN*
BMPR1A	2								
POLE	-	15	1		3				
MSH6	-	-	36	1	2				
MSH2	-	-	-	43	3		1		
MLH1	-	-	-	-	62				
APC	-	-	-	-	-	46			
POLD1	-	-	-	-	-	-	3		
PMS2	-	-	-	-	-	-	-	9	
PTEN	-	-	-	-	-	-	-	-	1

Supplementary Table 7: Occurrence of Class 3 co-mutations in cases for known CRC predisposition genes

Gene	*MSH6*	*MSH2*	*MLH1*	*BMPR1A*	*POLE*	*APC*	*SMAD4*	*POLD1*	*STK11*	*PMS2*	*PTEN*
MSH6	59	4	4		4	4		1			
MSH2	-	55	4		2	5		1			
MLH1	-	-	72	1	5	2	1	2			
BMPR1A	-	-	-	3				1			
POLE	-	-	-	-	40	4	1		1		
APC	-	-	-	-	-	94			1	1	
SMAD4	-	-	-	-	-	-	4				
POLD1	-	-	-	-	-	-	-	6			
STK11	-	-	-	-	-	-	-	-	6		
PMS2	-	-	-	-	-	-	-	-	-	12	
PTEN	-	-	-	-	-	-	-	-	-	-	2

Supplementary Table 8: Details of all Class 1 co-mutations for cases with Class 1 mutations in known genes

	Class 1 mutations in cases in known CRC genes					No cases	No controls
**All genes**	***MSH6***	***MLH1***	***APC***	***MSH2***	***PMS2***		
MSH2				31		31	0
MLH1		21				21	0
APC			18			18	1
MSH6	8					8	1
POLQ	1	2	1	1		24	33
SULT1C4	1	1	1	1		17	**25**
CDH26	1			3		14	18
COL6A5				3		43	54
KIAA0586		1	**1**	1		15	17
ANO5			1	1	1	13	11
MICU2		1		2		13	16
RBM43		1		2		13	10
IFNA5	1			2		12	20
ZNF599	1		1	1		9	23
IGSF10		1	1			24	23
ZSWIM1	1			1		23	23
FAM81B		1		1		23	18
USP45		1		1		20	28
ASAH2		1		1		19	26
GBP5		1	1			19	21
DSCR8				1	1	18	26
ACADL				2		17	21

CXCL6				2		17	20
OR6P1	1			1		16	9
ANKRD30A			1	1		16	29
ZC2HC1C			1	1		16	15
MSS51	1			1		15	15
HLA-G		2				15	20
SLC22A11	1		1			15	15
CCDC66			1	1		14	23
HHLA2		1		1		14	9
HABP2	1			1		14	18
CFHR5			1	1		13	34
IFIH1		1		1		13	18
SLFN12L	1	1				13	11
TRIM31	1	1				13	11
CARS2	1		1			13	13
FAM227B	1		1			13	8
DAPL1		1	1			13	26
TCHH			1		1	12	19
MLKL		1	1			11	24
SVOPL		1		1		10	14
ELMO3		1		1		10	15
TRIM38			1	1		10	9
CCR5		2				10	12
PITRM1			1	1		9	14
PSMB11		2				8	13
ERAP1				2		8	16
PYGM				2		8	15

NUDT13		1		1		7	18
IFNB1				2		7	7
CUBN			1	1		6	7
TRUB2	1	1				6	7
RIPK3			2			6	7
RP11-934B9.3			2			6	7
TMTC1	1			1		6	11
EDN3		1	1			6	6
EYS		1		1		6	4
ZNF165		2				5	4
OR2B2		2				4	4
CCDC14		1		1		4	5
STK31	1				1	4	7
KIAA1328	1		1			4	4
CEP135			1	1		3	1
ATP9B		1		1		2	2
NOSTRIN	2					2	3
OR6T1		1		1		2	1
PMS2					2	2	0
CFHR2				1		24	23
MROH2B	1					23	32
FAM71A			1			20	24
ENTHD1				1		17	31
PDE11A				1		17	30
SFXN3			1			16	17
TIAM2				1		16	14
FAM221A	1					16	28
ARL11				1		15	29

MYH15				1	14	18
ECHDC2			1		14	14
TP53AIP1			1		13	26
S100A3		1			13	16
CWH43		1			13	29
TIGD4				1	13	14
RGPD3			1		13	20
ABTB1			1		13	23
SMLR1		1			12	7
OR4F15				1	12	27
SERPINB10		1			12	12
ABCC11				1	11	8
PZP				1	11	24
TNFSF18				1	11	15
OR6F1				1	11	11
SPNS3	1				11	14
CARF			1		11	16
RP11-332O19.5			1		11	18
SNAPC1		1			10	16
DNAH7		1			10	23
WDR87	1				10	10
OSBPL1A			1		10	14
BORA		1			10	22
DYTN			1		10	9
PLA2G3			1		10	10
POLN			1		10	14
MYH8	1				10	7

AMZ2					1	9	14
TERF2IP				1		9	18
ZBED6CL				1		9	11
OR10C1				1		9	13
EGFL8		1				9	25
FANCL				1		9	14
WDR5B		1				9	13
AUNIP	1					9	28
THNSL1		1				9	8
SLC13A1			1			8	13
TRIM45				1		8	6
SIGLEC5			1			8	13
TMEM232		1				8	24
MMP10				1		8	21
GPR162				1		8	10
DUOX2		1				8	8
PPP1R3A				1		8	10
CD5L			1			8	12
HLA-B			1			8	26
PCDHA8		1				8	8
SLFN12		1				7	6
PCDHGA10				1		7	16
ZC3H8				1		7	18
SFI1	1					7	6
MUC7		1				7	4
CTSW					1	7	14
AFM	1					7	7

POLR3C		1			7	15
LY75-CD302		1			7	10
LY75		1			7	10
CYP2C18			1		7	10
PCM1		1			7	12
OVCH1	1				7	8
C5orf52			1		7	12
AQP7			1		7	12
KRTAP24-1			1		6	12
DNHD1			1		6	13
TRPA1		1			6	4
IGFN1				1	6	13
BRCA2	1				6	5
GCNT3				1	6	6
DNAH14				1	6	7
FBXW8				1	6	18
TAS2R10		1			6	1
ZRANB3				1	6	11
OPN4		1			6	15
CCDC175				1	6	10
PCDHB11				1	6	14
CYB561D2			1		6	16
OR51M1	1				6	6
MYO1A	1				6	10
CCDC105			1		6	17
DPEP2	1				6	15
SPATA33				1	6	17

TRIT1			1		6	2
RETSAT	1				6	7
MYH7B	1				6	8
WDR66			1		6	12
EMR1		1			5	0
DNA2			1		5	6
ASIC3			1		5	8
UGGT2		1			5	17
ASPG		1			5	1
RESP18				1	5	2
ROS1			1		5	4
CHMP4A		1			5	8
TM9SF1		1			5	8
MPPE1			1		5	8
DCD			1		5	6
MFSD6L	1				5	10
MICALCL				1	5	7
GPD2		1			5	6
TRIM59			1		5	9
XIRP2		1			5	19
SERPINB12		1			5	4
GCA			1		4	9
CCDC18				1	4	4
CASP5	1				4	8
ATP8B4		1			4	4
UPK2	1				4	8
CEP164				1	4	7

OR3A1		1				4	7
HIST1H4B				1		4	3
AAR2		1				4	8
MOCOS			1			4	5
C12orf74	1					4	7
STX10				1		4	5
DNAH8				1		4	7
GOT1L1				1		4	0
ZCCHC4			1			4	12
NUDT7		1				4	6
HCAR3			1			4	2
ZNF138			1			4	6
IRAK3		1				4	9
HAVCR1				1		4	4
DHFRL1		1				4	7
C9orf131			1			4	1
APOBEC3G				1		4	2
VPS13C				1		4	6
TPPP2			1			4	4
ZNF788				1		4	4
MTERF				1		3	7
TTC37		1				3	2
OR8D4					1	3	0
GBP7		1				3	8
ZNF284				1		3	4
SLC22A16				1		3	4
FANCC				1		3	4

ZNF439			1		3	2
ABCG8			1		3	3
C2orf53	1				3	6
ZNF44			1		3	4
CHRNA6	1				3	1
NFE2L3				1	3	1
ARSG				1	3	2
ADAM18				1	3	2
AC012215.1				1	3	0
CEACAM1				1	3	13
CBLC				1	3	4
BMP2K				1	3	3
DNAH6		1			3	7
PPP2R1B				1	3	2
COL4A3				1	3	1
MS4A6A			1		3	12
FNDC7	1				3	5
SPATA31E1			1		3	7
SPATA31D1		1			3	8
OR5F1				1	3	3
OR2G6		1			3	7
SHCBP1		1			3	3
SLC28A2			1		3	4
KIF6				1	3	2
GLB1L3		1			3	2
BRF2				1	3	1

SAMD9			1			3	3
NLRP14		1				3	2
TMC4				1		2	0
SLC15A2				1		2	0
OR8J1			1			2	2
C2CD3				1		2	3
ZNF556		1				2	0
C8G				1		2	1
BCHE		1				2	2
KLHL41		1				2	0
ATAD3B	1					2	3
NUDT12		1				2	1
ALDH1L2				1		2	5
EML5			1			2	0
SULT1A2				1		2	0
GDF9		1				2	1
OR8K3				1		2	5
SPIDR		1				2	2
KIAA1551					1	2	1
ERMARD		1				2	1
ADD1			1			2	0
PCDH15		1				2	2
MFI2		1				2	4
HUNK				1		2	0
C4orf45				1		2	0
IQGAP3				1		2	2
ALDH1L1				1		2	1
OBSL1		1				2	3

ZNF563	1					2	0
CCDC125				1		2	1
ANKMY1				1		2	1
SLC44A3				1		2	0
RTN2				1		2	0
PLCD1	1					2	2
NAAA			1			2	0
CTSE		1				2	1
FAM47E-STBD1		1				2	2
SYTL2		1				2	6
SEMG2			1			2	7
CETP			1			2	0
KIF13A				1		2	0
SUCO				1		2	2
PDCD1LG2			1			2	5
MYLK3	1					2	0
WRB		1				2	0
PHLDB2				1		2	0
CCDC178				1		2	0
KRT75				1		2	2
FAM129A		1				2	4
PROK2	1					2	3
EIF2B3					1	2	0
SLC35B2				1		2	0
C12orf50				1		2	1
PAG1					1	2	0
GZMK		1				2	0
DNAH1		1				2	5

PARP11		1			2	0
GPR133		1			2	1
TRAPPC2L				1	2	0
MAP10			1		2	1
OAS3			1		2	1
CLUL1				1	2	0
MYLK4			1		2	3
C10orf35				1	2	0
LIG4				1	2	2
OR7C1				1	2	2
PRUNE2				1	2	6
AGXT2		1			2	3
CDC7				1	2	2
WDR31			1		2	7
RNF207				1	2	0
EPPK1				1	2	2
KLHL33		1			2	7
GRIK1		1			2	1
COL28A1	1				2	3
MRPL39			1		2	0
PPIL2				1	2	1
GYPB				1	2	2
GADD45GIP1			1		2	1
OXGR1				1	2	4
PRR23A				1	2	1
FMO2		1			2	2
SLC29A2				1	2	3
PCDHB1		1			2	2

LILRA3				1	2	7
TUBGCP6		1			2	0
LGALS8				1	2	2
CEP44				1	2	0
AHNAK2			1		2	1
NPC1				1	2	1
IL11RA				1	2	0
ATF6B		1			1	0
XDH		1			1	0
PRADC1				1	1	0
PADI1				1	1	0
CAPNS2				1	1	1
BANK1	1				1	5
CASP6				1	1	3
DARS		1			1	1
STX8			1		1	0
UTRN		1			1	0
PDCD5				1	1	0
PLEKHG7				1	1	1
SLC24A5			1		1	0
METTL25		1			1	2
PDZD3			1		1	1
CRHBP				1	1	0
MTMR14				1	1	1
CCDC181		1			1	0
ANKLE2		1			1	0
METTL12				1	1	0
ATP10A			1		1	0

SOWAHB				1		1	0
CTC-554D6.1			1			1	0
NPFFR2				1		1	1
WDR96		1				1	2
ARHGEF26			1			1	1
C2orf61				1		1	0
FAM161A			1			1	2
EPS8L3			1			1	0
CYB5R4				1		1	0
IGFLR1		1				1	8
RRAS				1		1	0
PCDHA12		1				1	0
DSC1				1		1	0
CEP72				1		1	0
MCTP2				1		1	3
LMX1A				1		1	0
FBXO7	1					1	2
TRIP11			1			1	4
SLCO1C1		1				1	0
PCDHB6		1				1	0
CDH3				1		1	0
FIG4		1				1	1
ASNA1				1		1	0
PARP15		1				1	4
ME2					1	1	1
TMED3				1		1	0
HTR4			1			1	0
PRPS1L1				1		1	2

EFHD2				1		1	0
OR5L2			1			1	1
MYLK			1			1	0
OR51B2					1	1	0
ADAMTS8			1			1	0
SNX11		1				1	0
F2R			1			1	0
PTPRG			1			1	0
PRDM10				1		1	0
KIF27			1			1	0
CAPS2				1		1	0
GUSB				1		1	0
ANKLE1					1	1	0
VCAN			1			1	0
ZDHHC21				1		1	0
PCDHGA11		1				1	2
LOXL4			1			1	0
SULF1		1				1	0
KATNAL2				1		1	0
ELTD1			1			1	1
DCHS1			1			1	0
NSUN7	1					1	2
GALNT1				1		1	0
C9orf3		1				1	1
SYNM				1		1	3
TRMT5		1				1	3
CPSF2		1				1	0

BAIAP2L1			1			1	1
CLPB			1			1	0
USP29				1		1	1
SENP1		1				1	0
CAPN3				1		1	0
KCNAB3				1		1	0
TPD52L2	1					1	0
RFC3			1			1	0
LAMP1	1					1	0
NEDD4			1			1	0
RASAL1	1					1	1
THBS3			1			1	0
ANKAR	1					1	8
GPCPD1			1			1	1
AKR1C2				1		1	0
ZNF527			1			1	0
RIPK2		1				1	0
ARHGAP28		1				1	0
CARD6				1		1	2
STEAP4		1				1	0
AOC3				1		1	1
CPSF3		1				1	1
ITPR1					1	1	0
AATF	1					1	3
DHX34			1			1	0
DIS3			1			1	3
RSBN1L		1				1	0

MED23				1	1	1
KRT2		1			1	0
MRPL43				1	1	2
DNMT3L				1	1	0
CD200R1L		1			1	0
CYP4X1				1	1	2
FAP				1	1	0
MYOM2			1		1	6
PRR21			1		1	0
OR5B12		1			1	1
T				1	1	0
SLC15A1		1			1	1
NPC1L1		1			1	0
TUBGCP2				1	1	1
NUTM1				1	1	2
DEFB132	1				1	0
ACSM4			1		1	1
CKAP2			1		1	1
NEMF				1	1	1
PTH2R				1	1	0
SEMA3D				1	1	1
OGFR		1			1	0
SLC47A2		1			1	3
ETAA1		1			1	1
GFAP			1		1	0
KIF24			1		1	0
TCF3				1	1	0
C18orf25			1		1	1

SLC26A5	1					1	0
CD300LF				1		1	0
OR5V1		1				1	1
RAD51AP2				1		1	4
RP1L1	1					1	1
SYT14					1	1	1
C1orf192				1		1	1
GPX3			1			1	1
CGN		1				1	0
ZZZ3			1			1	0
SNRNP40		1				1	1
RP11-432B6.3			1			1	0
IRAK4		1				1	5
PCDHGA6			1			1	0
TMEM168				1		1	1
POC1B				1		1	0
TRAF3IP1		1				1	0
ATMIN		1				1	0
SEMA3C		1				1	0
ANXA11				1		1	0

Supplementary Note 1: The co-inheritance of mutations in the known CRC genes

We identified on average 7, 73 and 182 Class 1, 2 and 3 mutations per sample with no significant difference between case and control distributions (p=0.24, 0.95 and 0.11 respectively). As our power to investigate epistatic relationships is low, we limited our analysis to the set of known CRC genes: *MLH1, MSH2, MSH6, APC, PMS2, POLE2, POLD1, STK11, SMAD4, PTEN* and *BMPR1A*. After adjusting for multiple testing we were unable to identify any significantly co-mutated genes in the following sets: Class 2 variants in known genes x Class 2 variants in known genes (Supplementary Table 6), Class 3 variants in known genes x Class 3 variants in known genes (Supplementary Table 7) and Class 1 variants in known genes x Class 1 variants in all genes (Supplementary Table 8).
